# Biomechanical differences between novice and experienced runners: a systematic review

**DOI:** 10.3389/fspor.2026.1733815

**Published:** 2026-02-23

**Authors:** Haisheng Shen, Zisheng Jin, Zihui Ma, Hongying Wang

**Affiliations:** 1 School of Physical Education, Shanghai University of Sport, Shanghai, China; 2 School of Athletic Performance, Shanghai University of Sport, Shanghai, China

**Keywords:** dynamic stability, gait variability, injury risk factor, kinematics, kinetics, novice runners, proximal control/coordination, running experience

## Abstract

**Background:**

Running offers substantial physical and psychological health benefits, however, novice runners experience a markedly higher incidence of running-related injuries than experienced runners. Differences in biomechanical characteristics between these groups contributed to this elevated risk, but findings remain inconsistent and need to be synthesized. This study aims to synthesize evidence on biomechanical differences between novice and experienced runners.

**Methods:**

A systematic search was conducted to identify studies published between 2004 and December 2025. Fourteen eligible studies (*n* = 457 runners) were included for analysis. Data were extracted on study characteristics, definitions of running experience, biomechanical variables, and the risk of bias was assessed using the NIH tool.

**Results:**

Included studies consistently reported that novice runners exhibited greater spatiotemporal variability, larger joint ranges of motion along with weaker proximal muscle control, poorer coordination, and lower dynamic stability compared with experienced runners. However, inconsistencies were observed in knee kinematic findings, particularly in knee flexion–extension and ankle motion patterns.

**Conclusions:**

Novice runners exhibit less stable and less coordinated gait patterns, reflecting reduced neuromuscular control and higher injury susceptibility. Standardized, multidimensional definitions of running experience and more rigorous biomechanical protocols are needed to clarify these mechanisms and guide targeted injury-prevention strategies.

**Systematic Review Registration:**

https://www.crd.york.ac.uk/PROSPERO/display_record.php?RecordID=607126, identifier CRD42024607126.

## Introduction

Running is one of the most widely practiced physical activities worldwide, offering substantial cardiovascular, metabolic, and psychological benefits (1, 2). It reduces the risk of all-cause and cardiovascular mortality, enhances mood and quality of life, and supports weight management and chronic disease prevention (1). Despite these well-documented advantages, only a small proportion of individuals maintain long-term participation. Many runners discontinue or reduce their running frequency within months of initiation (3), and running-related injuries (RRIs) are considered as a major reason for this dropout (3, 4). A one-year follow-up study reported that the annual incidence of RRIs can be as high as 53.1% (5). Notably, novice runners experience a substantially higher risk of injury than experienced runners, with nearly 48% ceasing running because of injury (6, 7). This elevated injury risk among beginners has negative consequences for individuals but also places strain on public health systems as it detracts from the protective health benefits associated with regular PA (8). Therefore, reducing injury risk in novice runners represent critical priorities for both practice and research.

Improper training load management, such as rapid increases in weekly mileage or intensity and insufficient recovery, along with factors including previous injury history, body composition, age, sex, footwear, and running surface, have been identified as key contributors to injury susceptibility in novice runners (9, 10). In recent years, several studies have further suggested that biomechanical parameters [e.g., step frequency, stride length, joint angles, joint moments, and ground reaction forces (GRF)] also play important roles in injury risk (11–13). A growing number of studies have compared biomechanical differences between novice and experienced runners (14, 15). For instance, runners with more years of running experience generally demonstrate higher cadence and shorter stride length (16, 17). However, no systematic review to date has synthesized the evidence linking running experience with biomechanical variables, which limits our understanding of the mechanical and behavioral bases underlying the higher injury rates observed in novice runners.

Another important issue concerns the inconsistent definition of “novice runners.” Some studies have defined running experience by years of running (16), whereas others have used weekly running volume as the criterion (17). Such definitional heterogeneity introduces conceptual bias and hampers cross-study comparability and interpretability.

To address these research gaps, this systematic review synthesises current evidence on the relationships between running experience and biomechanical variables, with the aim of clarifying the biomechanical characteristics that distinguish novice from experienced runners. By systematically categorising and comparing the diverse operational definitions of “running experience” used across studies, this review characterises the biomechanical profiles associated with each definition and elucidates potential methodological sources of inconsistency in the existing literature. These insights may inform the development of more tailored training programmes and injury-prevention strategies for novice runners.

## Methods

### Study design

The review protocol was prospectively registered in PROSPERO (Registration ID: CRD42024607126). This review is reported according to the Preferred Reporting Items for Systematic Reviews and Meta-Analyses (PRISMA) 2020 statement guidelines (18).

The search strategy was structured using the Observational Study PECO framework (Participants, Exposure, Comparison, Outcome, Study design) (19). Search strategy can be found in Table 1. One of the authors (anonymized for review) conducted a primary search in PubMed. Then, the search strings were translated for Web of Science, and EBSCO. Grey literature was excluded. The search covered the period from January 1, 2004, to 31st December, 2025. No language restrictions were applied. Detailed search string design and search results are available in Supplementary File S1.

**Table 1:** PECO question and study design inclusion and exclusion criteria.

Question component	Inclusion criteria	Exclusion criteria
Participants	Runners	No runners (e.g., power athletes, rugby player, soldier)
Exposure	Novice runners (i.e., the study used a variable to define runners’ running experience, such as “running years”)	No definition of runner's running experience
Comparison	At least one group with a non-novice level.	No comparison group with different running experience (e.g., female runners compared to male runners)
Outcome	Biomechanical factor	No biomechanical factor (e.g., cardiovascular)
Study Design	Observational cohort, cross-sectional study	Interventional studies, reviews

All articles identified through the search were exported into NoteExpress reference management software [NoteExpress Reference Management Software (Version 3.9). Beijing Aegean Software Company, Beijing, China.], where automated duplicate detection and removal were performed. Following de-duplication, two independent reviewers systematically screened the titles, abstracts, and full texts of all remaining records to determine their eligibility for inclusion in the review. Any discrepancies between reviewers were resolved through discussion until consensus was reached, and a third reviewer was consulted when necessary.

The screening and selection process was conducted according to predefined eligibility criteria. Articles were included if they met all of the following conditions: (a) written in English; (b) observational cohort or cross-sectional study design; (c) reported at least one biomechanical outcome variable; (d) included both novice and non-novice (experienced) runners for comparison; and (e) the primary physical activity under investigation was running. Studies were excluded if they met any of the following criteria: (a) not written in English; (b) randomized controlled trials or intervention-based studies; (c) did not include analysis of biomechanical variables; (d) did not compare novice and non-novice runners; or (e) the participants were engaged in physical activities other than running.

### Data extraction and synthesis

Two investigators independently extracted data from the included studies. For each included study, first author, publication year, participant characteristics, experimental procedures and conditions, biomechanical variables, measurement instruments and parameters, and main findings were extracted.

For the definition of running experience, when multiple indicators were employed to categorize runners’ experience levels, the variable that was consistently applied across all groups was extracted. For instance, if both “running years” and “weekly running volume” were used to define experienced runners, but only “running years” was applied to define novice runners, then “running years” was regarded as the operational measure of running experience within that study.

Given the substantial heterogeneity in study designs, outcome measures, and definitions of running experience, a formal meta-analysis or statistical subgroup analysis was not feasible. Therefore, heterogeneity was explored using a structured narrative synthesis approach, in line with recommendations from the BMJ Synthesis without meta-analysis (SWiM) in systematic reviews: reporting guideline (20) and Cochrane Handbook for Systematic Reviews of Interventions (21). Findings were organised and compared according to hypothesised methodological modifiers, including the definition of running experience, running environment (treadmill vs. overground), and key experimental conditions.

### Risk of bias

Risk of bias (RoB) was independently assessed by two investigators using the National Institute of Health (NIH) observational cohort and cross-sectional Study Quality Assessment tool (22). This tool is widely accepted for assessing the potential bias in observational cohorts and cross-sectional studies.

The scale included 14 items. Item#10 [Was the exposure (s) assessed more than once over time?] and Item#13 (Was loss to follow-up after baseline 20% or less?) are removed because of their lack of applicability to specific types of studies. The original score for high-quality research is “≥11” (42). After removing 2 unrelated items, the score for high-quality research in this review should be “≥9.” Two investigators assessed studies independently, resolving disagreements by consensus or third-party adjudication.

## Results

**Figure 1:**
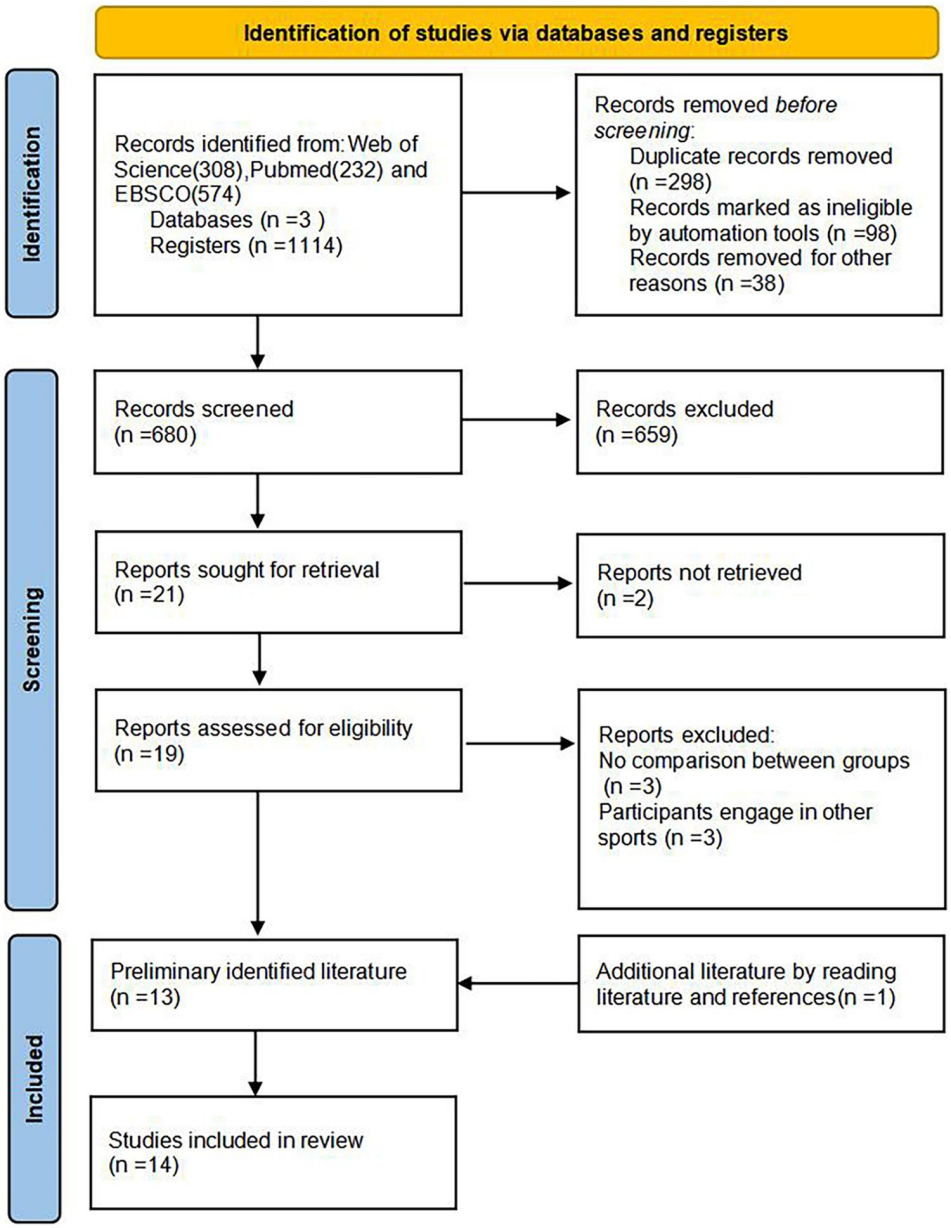
PRISMA flow diagram of literature selction process.

The systematic search yielded 1,114 records. After automatic duplicate removal, and manual screening, 13 articles were initially identified to be included in this review. Additional 1 article was added from literature reference list. Ultimately, a total of 14 articles were included in this review. PRISMA flow diagram are shown in Figure 1.

### Study characteristics

The characteristics of the included studies are summarized in Table 2. In total, the reviewed studies involved 457 runners. Among these, eight studies examined kinematic variables, five assessed kinetic variables, two investigated spatiotemporal parameters, five focused on coordination, symmetry, and stability, and two analyzed muscle activity.

**Table 2:** Characteristics of included studies.

Study	Participant	Participant characteristics	Indicators for defining “experience”	Running venue condition	Variables
Agresta et al. (16)	Size:38	(10*NOV) ≤ 3 years	Running years	Treadmill	Spatiotemporal
	Age: (EXP) 39.7 ± 9.7	(11*MED)4–9 years			
	(MED) 30.0 ± 11.0	(17*EXP) ≥ 10 years			
	(NOV) 34.9 ± 8.5				
Fadillioglu et al. (23)	Size: 25(♂)	(13*EXP) ≥ 2 years in club, ≥50 km/week	Running years	Treadmill	Spatiotemporal
	Age: (EXP) 23.5 ± 3.6	(12*NOV) <1year in club			
	(NOV) 23.9 ± 3.8				
Floría et al. (17)	Size: 22(♀)	(10*EXP) ≥ 35 km/week	Weekly running volume	Treadmill	Coordination
	Age: (EXP) 23	(12*NOV) <20 km/week			
	(NOV) 24 ± 4				
Frank et al. (24)	Size: 24 (♂)	(12*EXP) ≥ 30 km/week	Weekly running volume	Treadmill	Dynamic stability
	Age: (EXP) 23.3 ± 5.82	(12*NOV)no running habit			
	(NOV) 21.5 ± 2.71				
Hafer et al. (25)	Size: 41	(20*EXP) ≥ 10 years	Running years	Treadmill	Coordination
	Age: (EXP) 38.2 ± 10.9	(21*NOV) ≤ 2 years			
	(NOV) 32.3 ± 9.4				
Harrison et al. (7)	Size: 20(♀)	(10*EXP) ≥ 1year	Running years	Treadmill	Kinematic
		(10*NOV)no running habit			
Jiang et al. (26)	Size: 30 (♂)	(15*EXP) ≥ 30 km/week	Weekly running volume	Overground	Kinematic
	Age: (EXP) 23.65 ± 1.67	(15*NOV)2–10 km/week			Kinetic
	(NOV) 23.8 ± 1.97				
Kim et al. (43)	Size: 20	(10*EXP)participated in either a half or full marathon in 3 years	Competition experience	Treadmill	Kinematic
	Age: (EXP) 31.2 ± 9.8				Kinetic
	(NOV) 29.3 ± 6.8	(10*NOV)never taken part in competitions			
Maas et al. (27)	Size:30	(15*EXP) ≥ 50 km/week-♀, ≥70 km/week-♂	Weekly running volume	Treadmill	Kinematic
	Age: (EXP) 22 ± 4	(15*NOV) <10 km/week			
	(NOV) 21.1 ± 1				
Mo et al. (28)	Size: 34	(17*EXP) ≥ 4 years	Running years	Treadmill	Kinematic
	Age: (EXP) 24.9 ± 6.4	(17*NOV) < 6months			Coordination
	(NOV) 23.8 ± 4.7				
Mo et al. (29)	Size:31	using World Masters Association Age Grading Performance Tables	Running years	Treadmill	Kinetic
	Age: (EXP) 31.7 ± 4.1	(11*EXP) scores greater than 60% age-graged runners			Symmetry index
	(MED) 35.2 ± 7.4	(9*MED) scores less than 60% age-graged runners			
	(NOV) 29.1 ± 4.3	(11*NOV) <1year			
Quan et al. (30)	Size: 24 (♂)	(12*EXP) ≥ 20 mi/week, ≥i/week	Weekly running volume	Overground	Kinematic
	Age: (EXP) 26.2 ± 4.1	(12*NOV) 2–5 mi/week			Kinetic
	(NOV) 25.6 ± 4.7				Muscle activity
Schmitz et al. (31)	Size: 38 (♀)	(19*EXP) ≥ 1year	Running years	Treadmill	Kinematic
	Age: (EXP) 23 ± 3	(19*NOV) no running for at least 5 years			Kinetic
	(NOV) 24 ± 3				Muscle activity
Suda et al. (32)	Size: 78	Using a fuzzy decision-support system(total:10)	Running years & Weekly running volume	Treadmill	Kinematic
	Age: (EXP) 41.8 ± 7.0	(NOV) x < 5			
	(MED) 40.6 ± 7.1	(MED)5 ≤ x ≤ 7			
	(NOV) 40.1 ± 5.3	(EXP)x > 7			

EXP, experienced runner; NOV, novice runner; MED, medial experienced runner; ?, fail to use a common metric to distinglish novice runners from other runners.

The definition of “novice runner” varied across studies. Seven studies distinguished novice and experienced runners based on years of running participation, five used weekly running volume, one applied a combination of both criteria, and one classified participant according to competition experience.

Regarding experimental settings, 12 studies (86%) conducted tests on a treadmill, whereas two studies (14%) employed overground running. Control for potential confounding variables also differed across studies (Table 3). Specifically, only two studies (13%) controlled for participants’ foot strike pattern, while five studies (33%) standardized footwear conditions, of which one required participant to run barefoot, and four instructed them to wear identical running shoes.

**Table 3:** Process and result of included studies.

Study (Year)	Experimental equipment and parameters	Foot strike	Running equipment	Speed	Process and condition	Results (* = *p* < 0.1,** = *p* < 0.05)
Agresta et al. (16)	(kinematics) 8*camera motion analysis-200 Hz	/	Same type of running shoe	/	Warming up (5 min)	(NOV) stride length*↓, baseline stride time*↑, stride rate*↓
	(GRF)-force plate-1,000 Hz				→running through a laboratory (15 m)	
Fadillioglu et al. (23)	(kinematics) 8*camera motion analysis-200 Hz	/	Same type of running shoe	3.35 m/s	Warming up (5 min)	(NOV) DF*↑
					→run on a	CV(osc_CoM)**, CV(SF)**, CV(DF)**↑
	(GRF)-force plate-1,200 Hz				forceinstrumented treadmill (2 min)	
	(Muscle activity) hand held dynamometer					
Floría et al. (17)	(kinematics) 11* Vicon MX cameras-200 Hz	/	/	10 km/h & 15 km/h	Warming up (6 min)	no significant difference on hip-knee coupling and knee-ankle coupling between NOV and EXP
					→running in 15 km/h (15 s)	
					→rest (2 min)	
					→10 km/h (1 min) & 15 km/h (1 min)	
Frank et al. (24)	(Kinematics) IMU-200 Hz	/	/	Self-selected speed	Run on a treadmill	(NOV) movement stability for ankle**↓, knee**↓, hip**↓
HAFER et al. (25)	(kinematics) 10* Vicon MX cameras-150 Hz	/	/	1.94 m/s	→run on a treadmill	(NOV) thigh-shank coupling**↓, shank-foot coupling**↓
					→fatigue	
					→run on a treadmill (1 min)	
Harrison et al. (7)	(kinematics) 8*infrared Vicon cameras-100 Hz	heel-strike	barefoot	/	Familiarisation (7 min)	(NOV) knee abduction**↑, knee internal rotation**↑
					→running on a treadmill (5 km)	(NOV) hip adduction**↓, contralateral pelvis*↑
	(GRF) 3*force platforms-1,000 Hz.					
	(plantar pressure) pressure platform-50 Hz					
Jiang et al. (26)	(Spatiotemporal&VGRF)pressure-sensing treadmill-120 Hz	/	/	Self-selected speed	Warming up (5 min)	(NOV) ankle RoM (inversion/eversion)*↑,
					→external perturbation trials	hip RoM (adduction/abduction) **↑,
						knee RoM (flexion/extension)**↓
					→run in silence (3 min)	(NOV) ankle inversion moment**↑, ankle internal rotation moment**↑, hip abduction moment**↑, hip extension moment**↓
						peak propulsive GRF**↑
Kim et al. (2021)	(kinematics) 8*infrared cameras Vicon-200 Hz	heel-strike	same type of running shoe	Self-selected speed	Warming up(10 min)	(NOV) no significant difference on kinematic or plantar pressure in barefoot running between NOV and EXP
	1*force plates-1,000 Hz					
					→5 km	
					→running through a laboratory (12 m)	
Mass et al. (44)	(kinematics) 8*infrared cameras Vicon-200 Hz	/	/	Self-selected speed	Warming up	(NOV) trunk flexion*↑, trunk rotation RoM*↑, peak pelvic anterior tilt**↑, pelvic rotation RoM**↑, hip flexion RoM**↑, knee extension RoM**↑.
	2*force plates in tandem position-1,000 Hz				→treadmill (30)	
					→10 step cycles	
Mo et al. (28)	(kinematics) wireless inertial measurement system (IMU)-±2,000 °/s; 200 Hz	/	/	Self-selected speed	Running on a treadmill (5 min)	(NOV) hip RoM*↑, knee RoM**↑
						(NOV) anti-phase motion during midstance*↑
						(NOV) pelvis-thigh coupling (in-phase motion)*↓, hip-knee coupling*↓, knee-ankle coupling*↓, thigh-shanku coupling coupling*↓
	(VGRF) pressure-sensing treadmill-120 Hz					
						(NOV) CV for hip-knee coupling*↓, CV for shank-foot coupling*↓
Mo et al. (29)	(kinetic) 1,000 Hz	/	/	8, 9, 10, 11, 12 km/h	Warming up (10 min)	(NOV) bilateral asymmetry for time to peak vertical GRF at 9 km/h, VALR at 10 km/h, VILR at 8 km/h*↑
					→run on treadmill (3 min for every speed)	
						(NOV) the SIs decreased in the beginning,then ↑* with speed up
Quan et al. (30)	(kinematics) 5*camera motion analysis-200 Hz	/	/	Self-selected speed	Running on a treadmill(30–45 s)	(NOV) ankle RoM (dorsiflexion/dorsiflexion)**↑, hip RoM (flexion/extension)**↑, knee RoM (flexion/extension)*↑.
						(NOV) ankle moment (plantarflexion)*↑, hip momen (extension)**↓
						(NOV) VILR**↓
Schmitz et al. (31)	(kinematics) Optotrak motion capture system-100 Hz	/	same type of running shoe	Self-selected speed	Warming up	(NOV) trunk side-plank endurance*↓
					→run on a treadmill (4 min)	
Suda et al. (32)	(kinematics) 5*camera motion analysis-200 Hz	/	/	2.68 m/s	Warming up	(NOV) dorsiflexion angle**↑,plantarflexion angles**↑
					→run on a treadmill	
	(Kinetic) force plates-1,000 Hz					

‘↑’, lager; ‘↓’, lower; EXP, experienced runner; Nov, novice runner; MED, medial experienced runner; RoM, range of motion; GRF, ground reaction force; VILR, vertical instantaneous load rate; VALR, vertical average load rate; CV, coefficient of variation; osc_CoM, the vertical oscillation of the center of mass; SF, step frequency; DF, duty factor.

### Age-related characteristics across included studies

Across the included studies, participant age varied substantially, both between and within studies (Table 2). Mean ages ranged from early adulthood (approximately 21–24 years) to middle-aged runners (>40 years), with several studies including age-heterogeneous samples or reporting age differences between novice and experienced groups. Notably, studies defining running experience by years of running often included older experienced runners compared with novice runners [e.g., (16, 25)], whereas studies using weekly running volume tended to recruit younger cohorts with relatively small age differences between experience groups [e.g., (26, 30)].

### Sex distribution across studies

Considerable heterogeneity was also observed in sex composition across studies (Table 2). Of the 14 included studies, five recruited male-only samples, three included female-only samples, and the remaining studies involved mixed-sex cohorts. Among studies with single-sex samples, biomechanical differences between novice and experienced runners were broadly consistent with findings from mixed-sex studies, particularly for joint ranges of motion, coordination, and stability-related outcomes.

### Risk of bias assessment

The results of the risk of bias assessment are summarized in Table 4. Most studies (9, 64%) did not justify their sample size, and none (14, 100%) incorporated blinding procedures in their design or data collection. In total, six studies (43%) were rated as high quality.

**Table 4:** Summary of risk of bias assessment for included studies.

Study	NIH Scale												
	1	2	3	4	5	6	7	8	9	10	11	12	Quality
Agresta et al. (16)	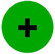	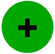	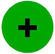	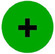	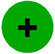	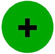	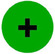	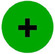	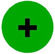	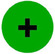	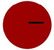	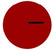	G
Fadillioglu et al. (23)	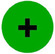	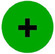	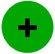	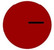	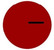	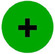	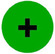	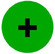	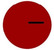	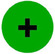	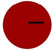	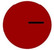	
Floría et al. (17)	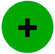	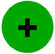	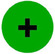	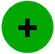	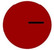	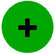	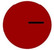	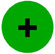	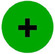	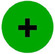	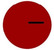	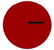	
Frank et al. (24)	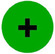	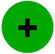	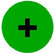	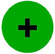	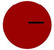	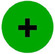	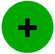	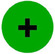	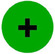	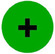	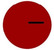	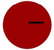	
Hafer et al. (25)	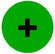	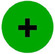	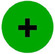	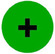	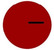	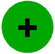	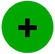	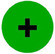	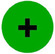	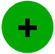	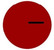	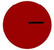	G
Harrison et al. (7)	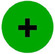	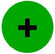	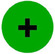	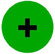	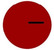	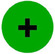	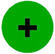	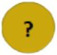	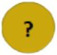	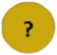	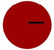	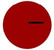	
Jiang et al. (26)	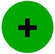	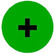	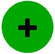	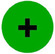	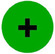	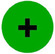	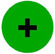	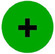	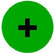	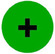	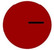	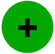	G
Kim et al. (43)	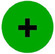	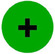	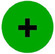	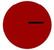	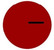	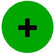	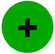	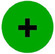	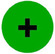	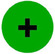	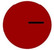	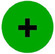	G
Mass et al. (44)	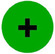	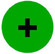	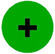	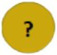	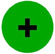	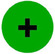	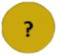	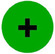	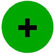	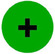	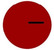	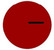	
Mo et al. (28)	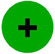	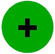	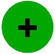	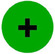	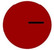	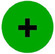	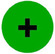	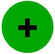	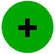	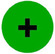	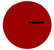	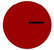	G
Mo et al. (29)	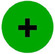	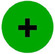	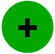	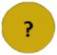	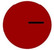	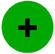	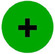	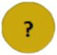	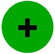	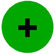	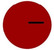	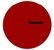	
Quan et al. (30)	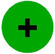	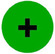	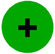	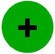	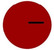	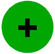	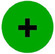	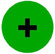	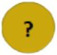	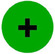	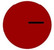	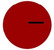	
Schmitz et al. (31)	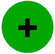	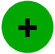	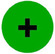	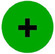	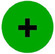	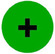	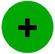	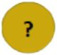	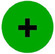	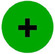	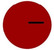	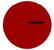	G
Suda et al. (32)	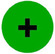	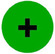	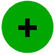	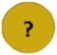	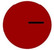	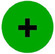	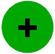	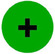	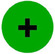	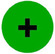	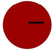	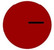	

(1) Clear question or objective? (2) Clearly specified and defined Population? (3) Participation rate of eligible persons at least 50%? (4) Subjects selected or recruited from the similar populations & Uniform inclusion and exclusion criteria? (5) Sample size justification? (6) Exposure of interest measured measured prior to the outcome being measured? (7) Timeframe sufficient to reasonably expect to see an association between exposure and outcome? (8) Examine different levels of the exposure? (9) Exposure measures clearly defined, valid, reliable, and implemented consistent? (10) Outcome measures clearly defined, valid, reliable, and implemented consistent? (11) Were the outcome assessors blinded to the exposure status of participants? (12) Key potential confounding variables measured and adjusted for their impact?.

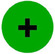
 = low risk bias, 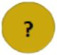 = unclear risk of bias, 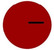 = high risk bias, G = high quality.

### The biomechanical characteristics of novice runners

The included studies defined runners’ experience based on running years, weekly running volume, or competition experience, and reported differences between novice and experienced runners in spatiotemporal, kinematic, kinetic, neuromuscular, and coordination-related variables (Table 2).

#### Spatiotemporal variables

Two studies investigated spatiotemporal variables among runners with different levels of experience. One study examined step rate, stride time, stride length, contact time, and flight time, and reported that novice runners with fewer running years exhibited lower stride length and step rate but longer stride time compared with experienced runners (16). The study also found that, when participants were required to adjust their step frequency, novice runners showed reduced ability to maintain the prescribed cadence compared with experienced runners. Similarly, the other study found that novice runners demonstrated greater variability, reflected by higher coefficients of variation (CV) in vertical oscillation of the center of mass, step rate, and duty factor (stance time/stride time) across different running speeds (23).

#### Kinematic variables

##### Ankle kinematics

Two studies that defined running experience by weekly running volume reported significant differences in ankle kinematics between novice and experienced runners. One study found that novice runners with lower weekly mileage exhibited greater ankle eversion angles (26). Another study found greater maximum plantarflexion and dorsiflexion angles among novice runners (30). Both studies were conducted in overground running environments, which are considered more ecologically valid than treadmill settings (33). Similarly, Suda et al. (32) classified runners using a combination of weekly running volume and running years, also reported larger ankle plantar/dorsiflexion angles in novice runners compared with their experienced counterparts.

##### Knee kinematics

Five studies investigated knee kinematics across running experience levels. Among the three studies defining experience by running years, significant differences were consistently observed. Harrison et al. (7) found that novice runners displayed greater internal rotation and knee adduction angles during the stance phase. In the sagittal plane, the other two studies reported larger knee flexion angles in novice runners (28, 29).

Another two studies defined experience by weekly running volume. One study found that novice runners demonstrated greater knee flexion angles (30), whereas the other reported the opposite trend, noting smaller flexion angles in the novice group (26).

##### Hip kinematics

Six studies examined the associations between hip kinematics and running experience. Three studies defined running experience by running years. Two studies reported larger hip internal rotation and abduction angles in novice runners (7, 31), while another study observed greater hip range of motion (RoM) in the sagittal plane (28).

The other three studies defined running experience using weekly running volume and similarly reported that novice runners exhibited greater hip RoM in both sagittal and coronal planes, with two of these showing statistically significant differences (26, 27, 30).

##### Trunk kinematics

Only one study examined the associations between trunk kinematics and running experience (27). With running experience defined as weekly running volume, the authors found that novice runners with lower weekly mileage exhibited greater trunk flexion peak and larger trunk rotational RoM compared with experienced runners.

#### Kinetic variables

##### Ankle kinetics

Two studies defined running experience as weekly running volume and examined the association between it and ankle joint kinetics. One study examining motion in the sagittal plane, reported that novice runners exhibited higher ankle plantar-flexion moments than experienced runners during the stance phase (30). Another study found that novice runners demonstrated greater ankle inversion and internal rotation moments (26).

##### Knee kinetics

Two studies defined running experience as weekly running volume and examined the association between it and knee kinetics. Both study found no significant differences in knee torques between novice and experienced runners (30) and (26).

##### Hip kinetics

Three studies examined hip joint kinetics. Two studies defined running experience as weekly running volume and reported consistent results that smaller hip extension moments and greater hip abduction moments among novice runners in the sagittal plane (26, 30). A third study defined running experience as running years also reported a reduction in hip extension moments among less experienced runners (15).

#### Ground reaction force and plantar pressure

Three studies assessed vertical loading rates. No group differences were detected in vertical average loading rate (VALR) when running experience was defined by either running years or weekly volume (29–31). However, one study reported a lower vertical instantaneous loading rate (VILR) in novice runners (30). Another study further found that novice runners with lower weekly mileage exhibited greater peak propulsive GRF (26). In contrast (15),observed no effect of experience on plantar-pressure distribution.

#### Muscle activity

Two studies examined the associations between muscle activity and running experience. One study defined running experience as lower weekly running volume have lower maximum hip muscle strength compared with experienced runners (30). Another study defined running experience as fewer running years found that novice runners have lower trunk muscle strength and endurance than experienced runners (31).

#### Coordination, stability and symmetry

Six studies examined lower-limb coordination, dynamic stability, and bilateral symmetry in runners of different experience levels. Two studies focused on hip–knee and knee–ankle coupling (17). found no significant differences between running experience in either coupling relationship. In contrast (28), defined running experience as running years, observed that novice runners exhibited lower hip–knee and knee–ankle coupling during the stance phase, as well as a lower coefficient of variation for coupling at each joint.

Two additional studies examined thigh–shank and shank–foot coupling, both defined running experience as running years. These studies similarly reported lower segmental coupling values among novice runners, indicating less coordinated lower-limb movement patterns (25, 28).

### Stability

One study investigated the associations between running experience and dynamic stability. This study found that novice runners with lower weekly running volume displayed reduced stability at the ankle, knee, and hip joints compared with experienced runners (24).

### Symmetry

Bilateral symmetry was evaluated in one study. This study reported that novice runners with fewer running years demonstrated poorer symmetry in GRF, VALR, and VILR (29).

### Investigation of heterogeneity across studies

To explore potential sources of heterogeneity in biomechanical findings, a structured narrative synthesis was conducted by stratifying studies according to hypothesised methodological modifiers, including the operational definition of running experience, running environment (treadmill vs. overground).

#### Heterogeneity by running experience

When studies were stratified by running experience, clear differences in the pattern of biomechanical findings were observed. Studies defining experience by running years more consistently reported differences in spatiotemporal variability, coordination, symmetry, and neuromuscular control, with novice runners showing shorter stride length, greater stride-to-stride variability, reduced inter-joint coordination, poorer bilateral symmetry, and lower trunk and hip muscle endurance. In contrast, studies defining experience by weekly running volume more consistently identified differences in joint kinematics and kinetics, with novice runners exhibiting larger joint ranges of motion at the ankle, hip, and trunk, altered joint moments, reduced dynamic stability, and greater asymmetry. Across both definitions, findings for knee kinematics were the most heterogeneous, with inconsistent directions of effect reported across studies. Evidence based on competition experience was limited to a single study and did not allow for systematic comparison.

#### Heterogeneity by running environment

Overground studies more consistently reported larger joint ranges of motion and altered joint moments at the ankle and hip in novice runners, whereas treadmill studies showed greater variability in kinematic outcomes, particularly for knee flexion–extension and ankle motion patterns. Variability in treadmill protocols, including differences in speed control, fatigue exposure, and footwear standardisation, further contributed to heterogeneity across treadmill-based studies.

#### Heterogeneity by fatigue and task demand

When studies were stratified by task demand, biomechanical differences between novice and experienced runners were more consistently observed in protocols involving fatigue, prolonged running, or external perturbations. Under these conditions, novice runners more frequently exhibited larger joint excursions, reduced inter-joint coordination, lower dynamic stability, and greater asymmetry. In contrast, studies employing short-duration, non-fatiguing running tasks showed more variable or null findings across several kinematic outcomes, particularly for knee and ankle motion.

#### Heterogeneity by risk of bias

Studies rated as higher quality more consistently reported differences between novice and experienced runners in spatiotemporal variability, coordination, stability, and proximal control–related outcomes. In contrast, heterogeneity in knee and ankle kinematic findings was observed across studies irrespective of overall risk-of-bias rating, suggesting that inconsistencies in these outcomes were not solely attributable to methodological quality. The absence of blinding and sample size justification across most studies may have contributed to uncertainty in effect estimates for specific biomechanical variables.

## Discussion

### Overview of the findings

This systematic review synthesized evidence from 14 studies that investigated biomechanical differences between novice and experienced runners, with particular attention to how these differences vary according to diverse operational definitions of “running experience”. Across studies, novice runners consistently exhibited greater variability and poorer control in spatiotemporal parameters, larger joint ranges of motion (especially at the ankle and hip), and higher joint moments across multiple planes at the ankle. Novice runners also demonstrated reduced hip and trunk muscle strength, weaker inter-joint coordination, lower dynamic stability, and greater bilateral asymmetry during running.

### Biomechanical characteristics differences across operational definitions of running experience

**Figure 2:**
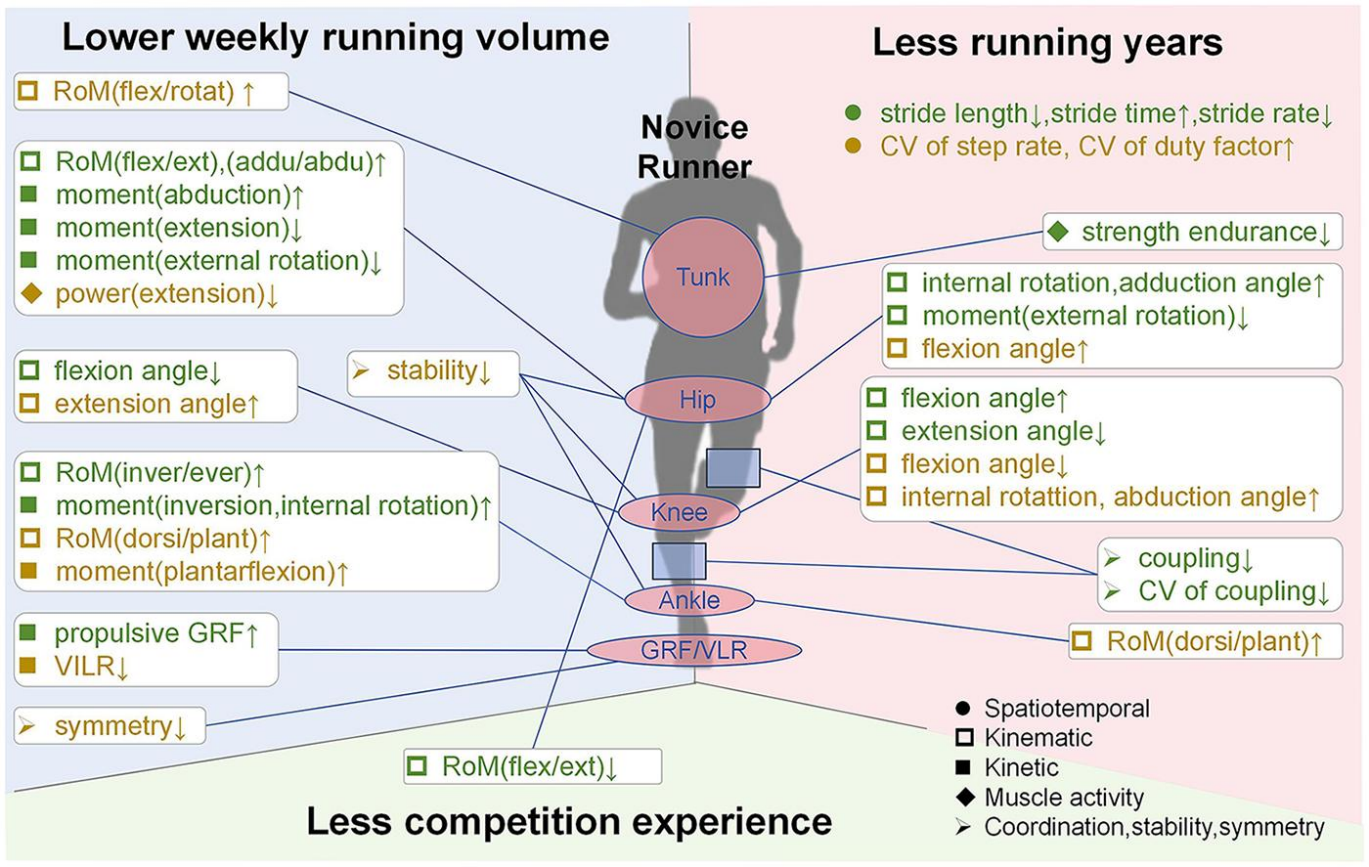
The biomechanics characteristics of novice runners with different definitions. The green font represents studies with high-quality. ‘↑’, lager; ‘↓’, lower; RoM, range of inotion; GRF, ground reaction force; VILR, vertical instantaneous load rate; CV, coefficient of variation; flex/ext, flexion/extension; dorsi/plan, dorsiflex/plantarflexion; flex/rotat, flexion/rotation; inver/ever, inversion/eversion.

The biomechanical characteristics of novice runners under different definitions are illustrated in Figure 2. In existing literature, *running experience* is operationalized as (i) years of running, (ii) weekly running volume (mileage), and (iii) competition or participation history. Across these definitions, novice runners exhibit broadly consistent biomechanical features. Specifically, runners with lower weekly mileage typically show increased joint ranges of motion in flexion–extension and rotation at the trunk and hip, reduced extension and external rotation moments, and diminished stability and symmetry. Those with fewer running years are characterized by shorter stride length, higher stride-time variability, weaker hip and trunk strength endurance, and inconsistent knee and ankle kinematics, whereas individuals with limited competition experience often display smaller joint excursions and lower propulsive ground reaction forces, indicating reduced propulsion efficiency and neuromuscular control.

Despite these consistent trends, discrepancies across studies are mainly concentrated in kinematic variables, particularly knee flexion/extension and ankle motion patterns, including variations in trunk rotation, hip internal–external rotation moments, knee flexion and extension angles, ankle dorsiflexion–plantarflexion and inversion–eversion ranges, stride length, stride rate, and coordination coupling. One possible explanation for this inconsistency is that “running years” primarily capture the long-term exposure and accumulated motor learning of running techniques, whereas “weekly running volume” reflects more recent training load, fatigue level, and current physical conditioning. These two metrics therefore represent different dimensions of running experience, one emphasizing chronic adaptation, the other acute training status.

### Interpretation on the biomechanical characteristics of novice runners

The review found that novice runners experience exhibited greater spatiotemporal variability and lower baseline stride rates, particularly when required to adjust their step frequency, compared with experienced runners. This difference may stem from insufficient neuromuscular coordination, stability, strength, and endurance among novice runners (34). Novice runners may rely more heavily on conscious motor control and less on automatized gait patterns, leading to inconsistent temporal regulation and stride variability. Limited exposure to repetitive loading cycles may also restrict the optimization of central pattern generators responsible for rhythmical locomotion, resulting in less stable and less economical gait control (34). Moreover, inadequate muscle strength and endurance, particularly in the hip stabilizers, knee extensors, and ankle plantar flexors, can reduce the ability to maintain consistent limb trajectories and control impact forces. For example, studies have shown that novice runners often present poorer joint stability across the hip, knee, and ankle (24), which may impair shock absorption and alter timing between stance and swing phases. Deficient proprioceptive acuity and delayed muscle activation further compromise joint stiffness regulation, causing increased contact times and step-to-step variability (24).

Novice runners generally exhibit a greater ROM of ankle, particularly larger inversion–eversion excursions and increased inversion and internal-rotation moments than experienced runners (26). These differences likely stem from two interrelated factors. Physiologically, limited training exposure may lead to weaker peri-ankle musculature and reduced neuromuscular control. This can result in diminished joint stiffness and delayed muscle activation timing, reducing the ability to dynamically stabilize the ankle during stance and push-off (35). Consequently, the ankle may display exaggerated motion as muscles respond reactively rather than pre-emptively to ground contact. Methodologically, the 5 km pre-test run in that study may have induced fatigue among novices whose average weekly mileage was only 2–10 km, thereby amplifying joint excursions during measurement. Excessive ankle eversion during stance has been linked to elevated risks of Achilles tendinopathy, anterior cruciate ligament strain, and increased tibial stress (36, 37). Combined with the longer ground-contact times often observed in less experienced runners, these features may heighten tibial and soft-tissue loading. Moreover, the greater eversion and internal-rotation moments likely reflect compensatory stabilization efforts, demanding increased activation of the tibialis anterior and posterior tibialis muscles (38), which may contribute to the reduced running economy characteristic of novice runners.

The hip joint plays a critical role in lower-limb kinematics and stability, and its dysfunction has been identified as a key contributor to impaired running performance and injury risk (39). Across included studies, novice runners consistently exhibit greater hip RoM during the stance phase, suggesting reduced joint stability compared with experienced runners. This increased RoM may result from strength imbalances between the hip abductors and adductors, which compromise pelvic control and lead to excessive hip motion (30). Additionally, insufficient endurance of the hip and trunk musculature among runners with lower training volumes may exacerbate fatigue-induced instability. With prolonged running, novices appear less able to maintain neuromuscular control, resulting in greater lateral sway and difficulty modulating hip motion to absorb impact efficiently (27, 28).

### Age- and sex-related differences in biomechanical patterns

When interpreted through the lens of age and sex, the biomechanical differences observed between novice and experienced runners appear to reflect partially distinct mechanisms. Age-related variability across studies was more closely associated with neuromuscular control, coordination, and stability-related outcomes. Studies involving older experienced runners tended to report more consistent differences in stride-to-stride variability, inter-joint coordination, and dynamic stability, suggesting that accumulated running exposure and age-related motor learning may jointly contribute to more stable and automatized gait patterns. In contrast, studies with younger cohorts and narrower age ranges more frequently reported differences in joint kinematics and kinetics, particularly at the ankle and hip, indicating that training status and recent mechanical loading may play a larger role when age-related adaptations are less pronounced.

In comparison, sex-related differences were more likely to influence the magnitude and direction of joint-specific kinematic and kinetic outcomes. Female-only studies more commonly reported greater frontal and transverse plane motions at the knee and hip, whereas male-only studies more frequently identified differences in joint moments, propulsion, and stability-related measures. These patterns align with known sex-based differences in pelvic morphology, lower-limb alignment, and muscle activation strategies during running.

### Suggestions for future studies

This review highlights several methodological limitations in the existing literature that warrant attention in future research. First, substantial inconsistency exists in the operational definition of “running experience.” This definitional heterogeneity undermines cross-study comparability and constrains the extent to which biomechanical findings can be meaningfully synthesized. Future research would benefit from adopting standardized, multidimensional definitions of running experience that integrate indicators such as years of running, training frequency, weekly mileage, and performance level. Second, biomechanical assessments were conducted under heterogeneous experimental environments, with some studies employing treadmill running and others using overground protocols. Given well-documented biomechanical differences between these conditions (33), future studies should either standardize testing environments or explicitly compare treadmill and overground running to enhance ecological validity and interpretability. Third, potential confounding variables were insufficiently controlled across studies. Only a small proportion accounted for foot-strike pattern or standardized footwear, despite strong evidence that both substantially influence running biomechanics (13, 40). Improved methodological rigor in controlling or reporting these factors is essential to reduce uncertainty in biomechanical comparisons across experience levels. In addition, this review identified a notable gap in the investigation of trunk kinematics and their interactions with lower-limb biomechanics. Although trunk motion has been shown to influence lower-extremity loading and coordination (13), few studies have examined these relationships specifically in novice runners. Future research incorporating trunk-related biomechanical variables alongside ground reaction force measures may contribute to a more comprehensive understanding of whole-body adaptations associated with running experience. Finally, despite a growing body of research examining biomechanical differences between novice and experienced runners, considerable heterogeneity persists in participant characteristics and analytical approaches. This heterogeneity complicates the interpretation of findings and cautions against strong translation into practice. Future studies should incorporate age- and sex-stratified analyses or explicitly model age–sex–experience interactions to better distinguish experience-related biomechanical adaptations from demographic influences and to improve the generalizability of conclusions.

## Limitations

This review has several limitations. First, gender is a known factor influencing running biomechanics (41); however, the included studies did not consistently report or analyze sex-specific differences. Future research should stratify analyses by gender to better capture biomechanical variations. Second, the review was limited to studies published in English and only searched for three databases, which may have led to language and publication bias. Third, due to substantial heterogeneity across studies, a meta-analysis could not be conducted. Future research should adopt standardized definitions and biomechanical assessment protocols to enable meta-analytic integration and strengthen the evidence base in this field. Moreover, several methodological limitations were identified across the included studies. Most studies did not provide a justification for sample size, and none incorporated blinding procedures, which may have increased uncertainty in effect estimates for certain biomechanical outcomes. Although a subset of studies was rated as high quality, these risk-of-bias issues should be considered when interpreting the findings. In addition, the lack of age-stratified analyses across included studies limited the ability of this review to distinguish biomechanical differences attributable to running experience from those potentially driven by age-related neuromuscular and musculoskeletal adaptations.

## Conclusion

Novice runners show greater gait variability, increased ankle and hip motion, and weaker proximal control than experienced runners, resulting in less stable and less economical mechanics. However, no consensus has been reached on knee kinematic and kinetic differences, largely due to inconsistent definitions of “running experience” across studies. Future studies should adopt standardized, multidimensional criteria and rigorous biomechanical assessments to enhance comparability and guide targeted injury-prevention strategies.

## Data Availability

The original contributions presented in the study are included in the article/Supplementary Material, further inquiries can be directed to the corresponding author.
