# Supplementary material for: Biomechanical differences between novice and experienced runners: a systematic review
Source: Front Sports Act Living. 2026 Feb 23;8:1733815. doi: 10.3389/fspor.2026.1733815 (PMC12968214; doi:10.3389/fspor.2026.1733815)
Supplement: Supplementary file 1 [file Supplementaryfile1.docx]

**Supplemental file 1 Search results**

**Total:** 1114

**Wos:** 308

**Pubmed:** 232

**EBSCO:** 574

**Search string design**

**WOS**

(AB=(novice) OR AB=(experience) OR AB=(beginner)) AND AB=(runner) AND (AB=(biomechanic) OR AB=(kinematic) OR AB=(kinetic) OR AB=(spatiotemporal) OR AB=(plantarflexion) OR AB=(dorsiflexion) OR AB=(rotation) OR AB=(inversion) OR AB=(eversion) OR AB=(extension) OR AB=(flexion) OR AB=(adduction) OR AB=(abduction) OR (AB=(joint moment) OR AB=(joint power)) OR AB=(peak pressure) OR AB=(impulse) OR AB=(ground reaction force))

**Pubmed:**

((novice) OR (experience) OR (beginner)) AND (runner) AND ((biomechanic) OR (kinematic) OR (kinetic) OR (spatiotemporal) OR (plantarflexion) OR (dorsiflexion) OR (rotation) OR (inversion) OR (eversion) OR (extension) OR (flexion) OR (adduction) OR (abduction) OR ((joint moment) OR (joint power)) OR (peak pressure) OR (impulse) OR (ground reaction force))

**EBSCO**

(novice OR experience OR beginner) AND runner AND (biomechanic OR kinematic OR kinetic OR spatiotemporal OR plantarflexion OR dorsiflexion OR rotation OR inversion OR eversion OR extension OR flexion OR adduction OR abduction OR (joint moment OR joint power) OR peak pressure OR impulse OR ground reaction force)
